# Supplementary material for: Molecular basis of TMPRSS2 recognition by Paeniclostridium sordellii hemorrhagic toxin
Source: Nat Commun. 2024 Mar 4;15:1976. doi: 10.1038/s41467-024-46394-6 (PMC10912200; doi:10.1038/s41467-024-46394-6)
Supplement: Supplementary file 1 — Supplementary Information [file 41467_2024_46394_MOESM1_ESM.pdf]

Supplementary Information

Molecular basis of TMPRSS2 recognition by *Paeniclostridium sordellii* hemorrhagic toxin

Ruoyu Zhou[1,2,3,4,#], Liuqing He[2,3,4,5,#], Jiahao Zhang[2,3,4,#], Xiaofeng Zhang[2,3,4], Yanyan Li[2,3,4], Xiechao Zhan[2,3,4,*], and Liang Tao[1,2,3,4,5,*]

[1]College of Life Sciences, Fudan University, Shanghai 200433, China

[2]Center for Infectious Disease Research, Westlake Laboratory of Life Sciences and Biomedicine, Westlake University, Hangzhou 310024, China

[3]Key Laboratory of Structural Biology of Zhejiang Province, School of Life Sciences, Westlake University, Hangzhou 310024, China

[4]Westlake Institute for Advanced Study, Hangzhou 310024, China

[5]Research Center for Industries of the Future, Westlake University, Hangzhou, 310024, China

[#]These authors contributed equally to this work.

*Corresponding to:

Xiechao Zhan, Ph.D. Email: zhanxiechao@westlake.edu.cn

Liang Tao, Ph.D. Email: taoliang@westlake.edu.cn

Supplementary Figures

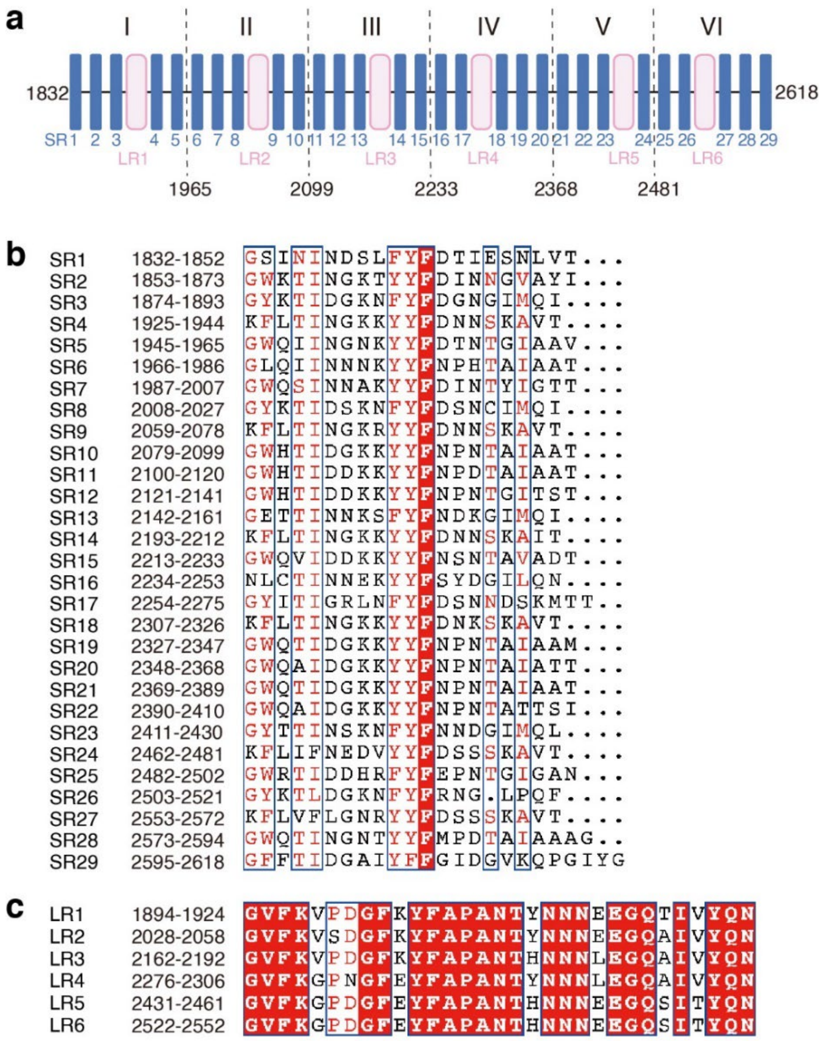

Supplementary Fig. 1 | Illustration of the TcsH CROP units, SRs, and LRs. a The schematic diagram showing the range of each TcsH CROP unit. **b-c** The range, and sequence alignment of TcsH-CROPs SRs (**b**) and LRs (**c**).

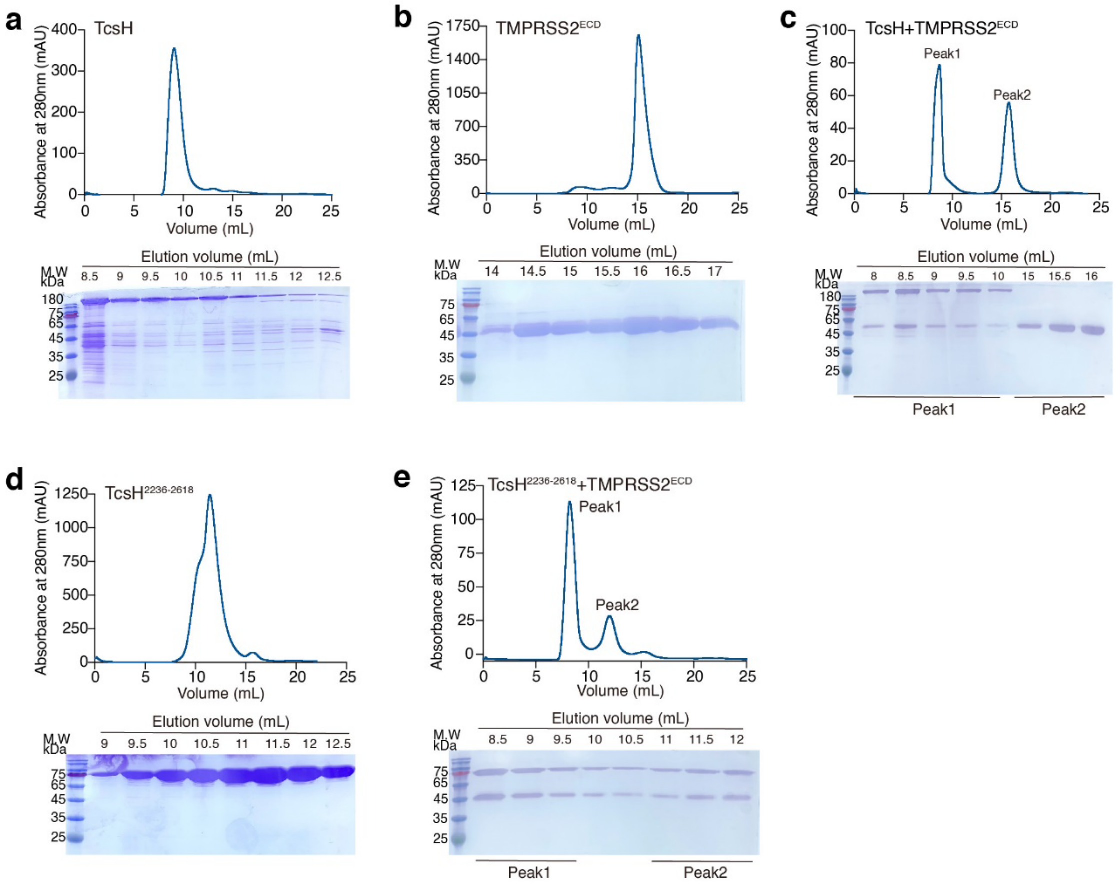

Supplementary Fig. 2 | Purification of proteins used for the cryo-EM study. a Size-exclusion chromatography profile of TcsH and SDS-PAGE of the peak fraction shown in the inset. **b** Size-exclusion chromatography profile of TMPRSS2^{ECD} and SDS-PAGE of the peak fraction shown in the inset. **c** The purified TcsH-TMPRSS2^{ECD} complex is eluted from gel filtration as a well-behaved peak (peak 1). **d** Size-exclusion chromatography profile of MBP-TcsH$^{2236\text{-}2618}$ and SDS-PAGE of the peak fraction shown in the inset. **e** The purified TcsH$^{2236\text{-}2618}$-TMPRSS2^{ECD} complex is eluted from gel filtration as a well-behaved peak (peak 2). The peak fraction was visualized on an SDS-PAGE gel and stained by Coomassie blue.

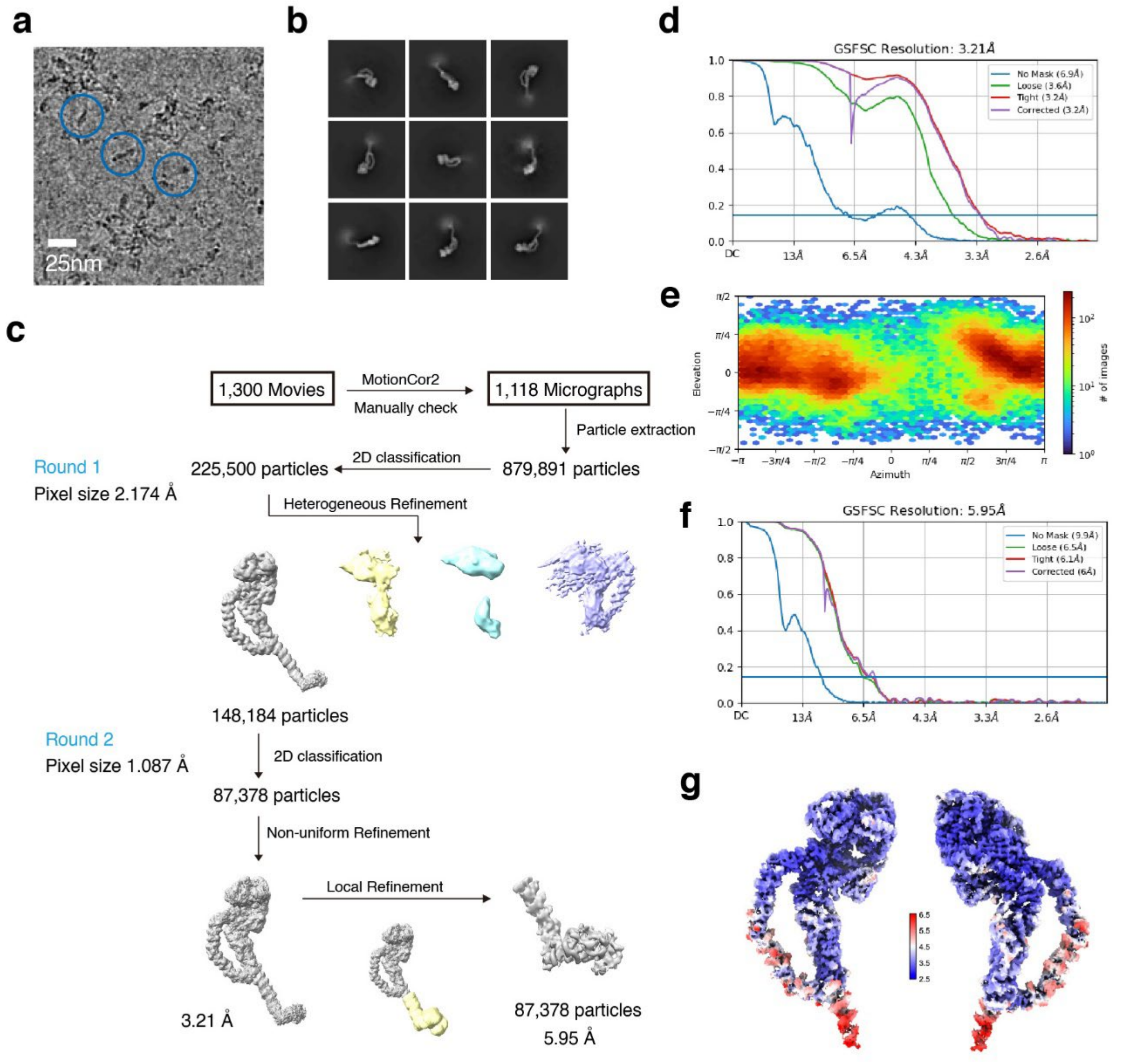

Supplementary Fig. 3 | Cryo-EM analysis of the TcsH-TMPRSS2^{ECD} complex. a A representative cryo-EM micrograph of the TcsH-TMPRSS2^{ECD} complex sample. **b** Representative 2D class averages of the TcsH-TMPRSS2^{ECD} complex sample. **c** A flow chart diagram of cryo-EM data processing for the TcsH-TMPRSS2^{ECD} complex. Please refer to the Methods section for details. **d** The final reconstruction of the TcsH-TMPRSS2^{ECD} complex has an average resolution of 3.2 Å as determined by the FSC value of 0.143. **e** Angular distribution of the particles in the final round of 3D refinement. **f** The average resolution of the overall EM map of the part around the TcsH C-terminus is estimated to be 6.0 Å. **g** Local resolution of the final cryo-EM map of the near-complete TcsH (residues 1-2387).

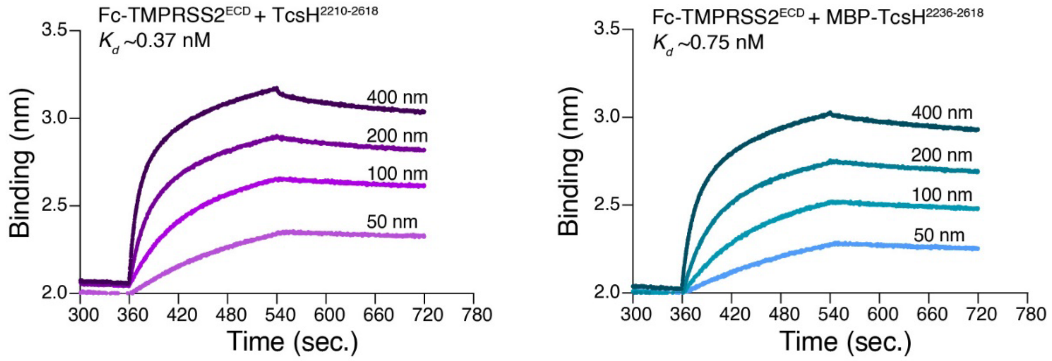

Supplementary Fig. 4 | Binding affinity of TcsH$^{2210\text{-}2618}$ and MBP-TcsH$^{2236\text{-}2618}$ to TMPRSS2^{ECD}. Representative binding curves of TcsH$^{2210\text{-}2618}$ (left panel) and MBP-TcsH$^{2236\text{-}2618}$ (right panel) to Fc-TMPRSS2^{ECD}. Source data are provided as a Source Data file.

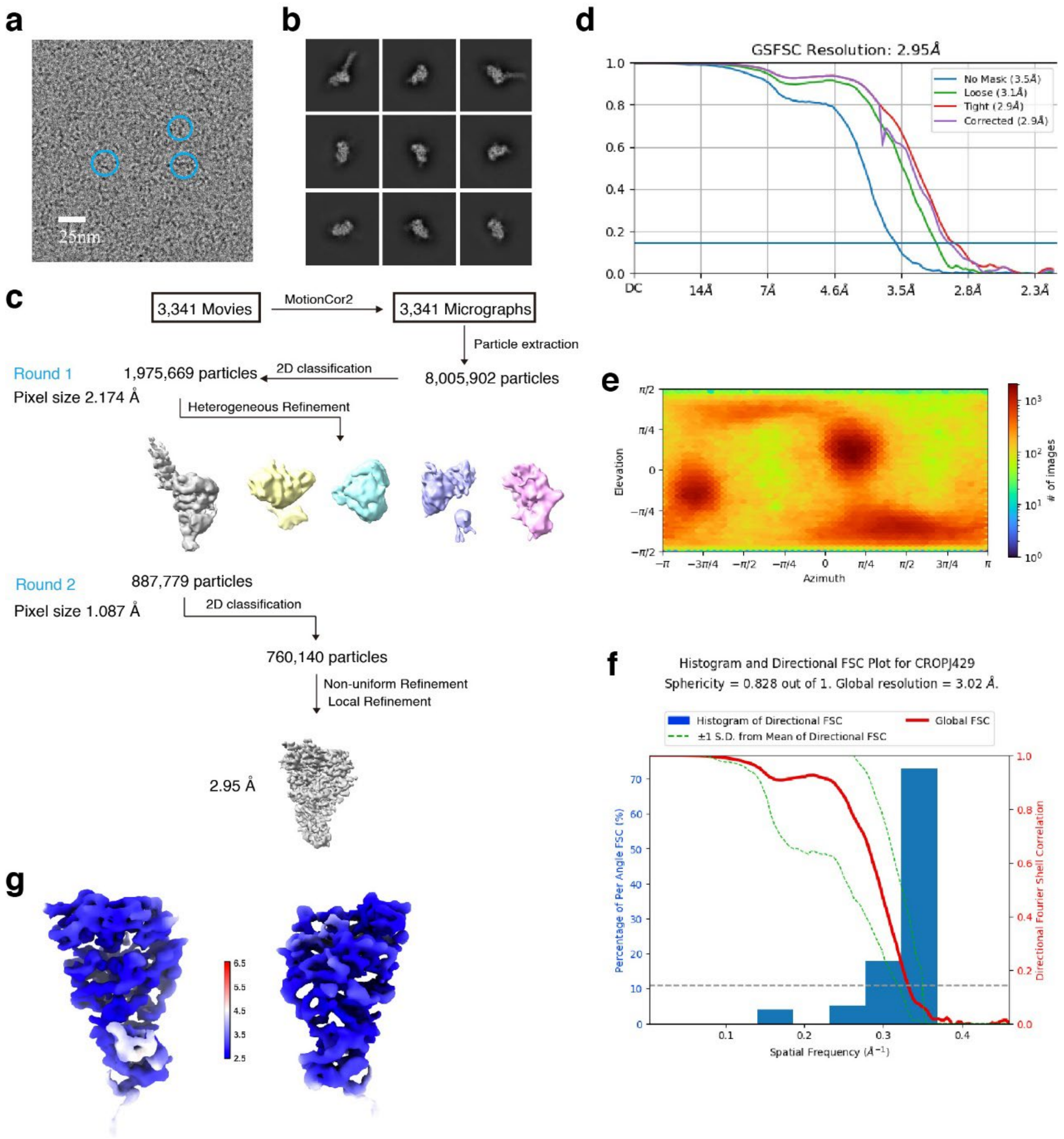

Supplementary Fig. 5│Cryo-EM analysis of the TcsH$^{2236\text{-}2618}$-TMPRSS2^{ECD} complex. **a** A representative cryo-EM micrograph of the TcsH$^{2236\text{-}2618}$-TMPRSS2^{ECD} complex sample. **b** Representative 2D class averages of the TcsH$^{2236\text{-}2618}$-TMPRSS2^{ECD} complex sample. **c** A flow chart diagram of cryo-EM data processing for the TcsH$^{2236\text{-}2618}$-TMPRSS2^{ECD} complex. Please refer to the Methods section for details. **d** The final reconstruction of the TcsH$^{2236\text{-}2618}$-TMPRSS2^{ECD} complex has an average resolution of 3.0 Å as determined by the FSC value of 0.143. **e** Angular distribution of the particles in the final round of 3D refinement. **f** Histogram and directional FSC plot for the EM map of TcsH$^{2236\text{-}2618}$-TMPRSS2^{ECD} complex. **g** Local resolution of the final cryo-EM map of TcsH$^{2246\text{-}2616}$ in complex with TMPRSS2^{ECD}.

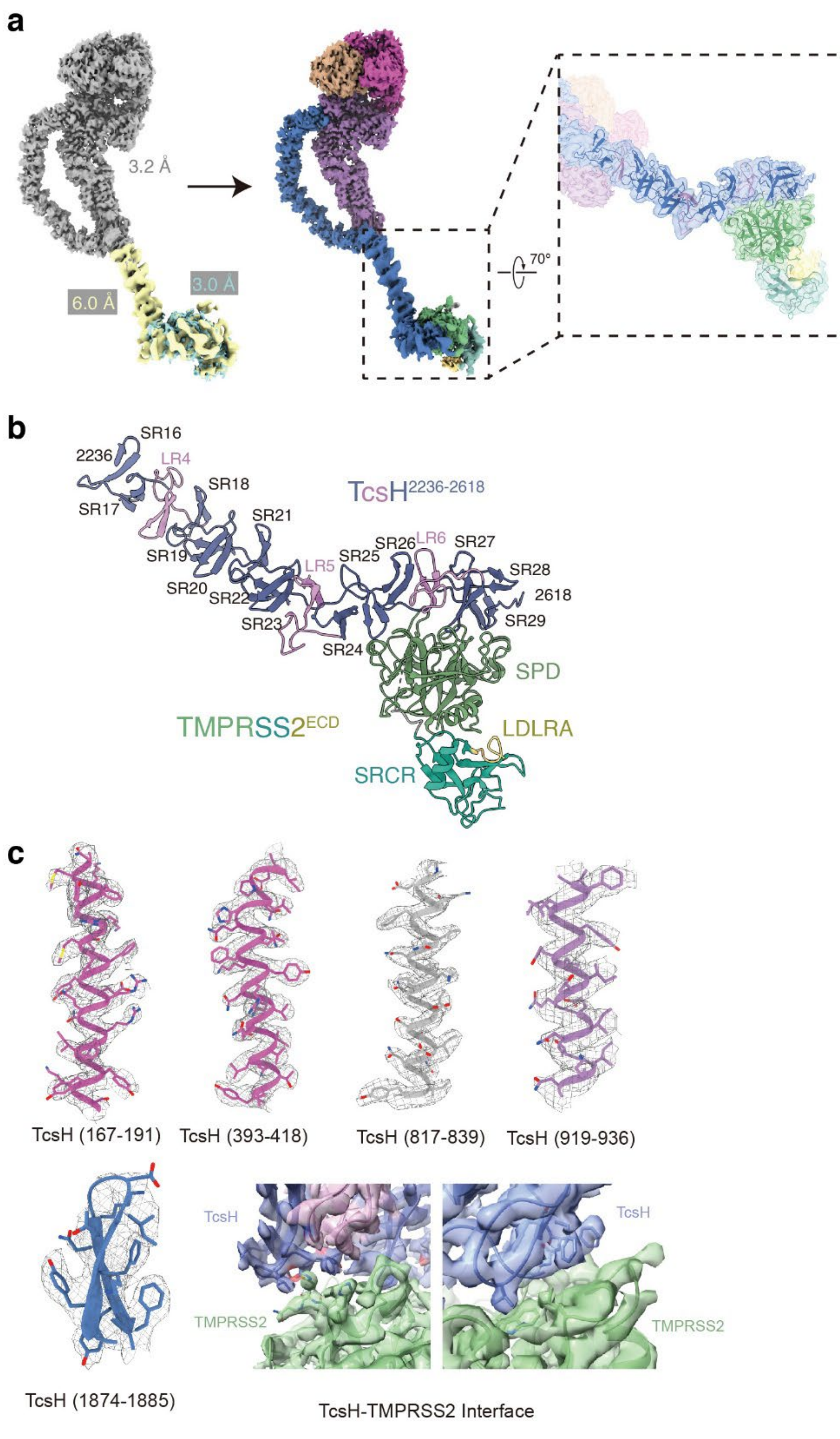

Supplementary Fig. 6 | The complete EM map of the TcsH-TMPRSS2^{ECD} complex. **a** The complete EM map of the TcsH-TMPRSS2^{ECD} complex is composed of a 3.2 Å map of the core region of TcsH (grey), a 6.0 Å local resolution map around the C-terminus of TcsH and a 3.0 Å EM map of TcsH$^{2236\text{-}2618}$-TMPRSS2^{ECD} complex fitted into the 6.0 Å EM map. **b** A cartoon representation of the TcsH$^{2236\text{-}2618}$-TMPRSS2^{ECD} complex. TcsH$^{2236\text{-}2618}$ is composed of SR16-29 and LR4-6. **c** Quality of the EM density map for selected regions of the model and the interfaces between TcsH and TMPRSS2 complex

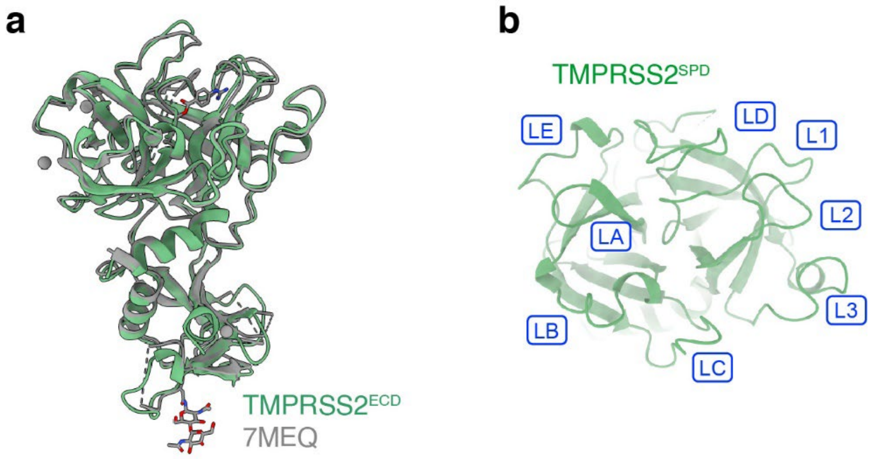

Supplementary Fig. 7 | The structure of TMPRSS2^{ECD}. **a** Structure comparison between our cryo-EM structure of TMPRSS2^{ECD} and a crystal structure of TMPRSS2$^{106-492}$ (PDB: 7MEQ). **b** A cartoon representation of TMPRSS2^{ECD} from the top view, eight surface loops are noted.

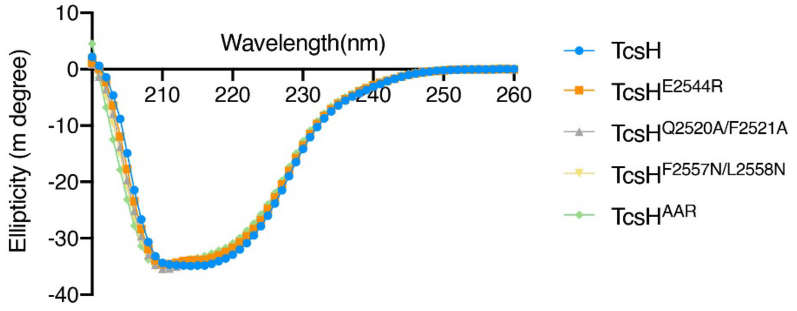

Supplementary Fig. 8 | CD spectroscopy of the WT and mutant TcsH. CD spectra were recorded in the wavelength range of 190 to 260 nm; the curves of the WT and mutant TcsH proteins are similar. Source data are provided as a Source Data file.

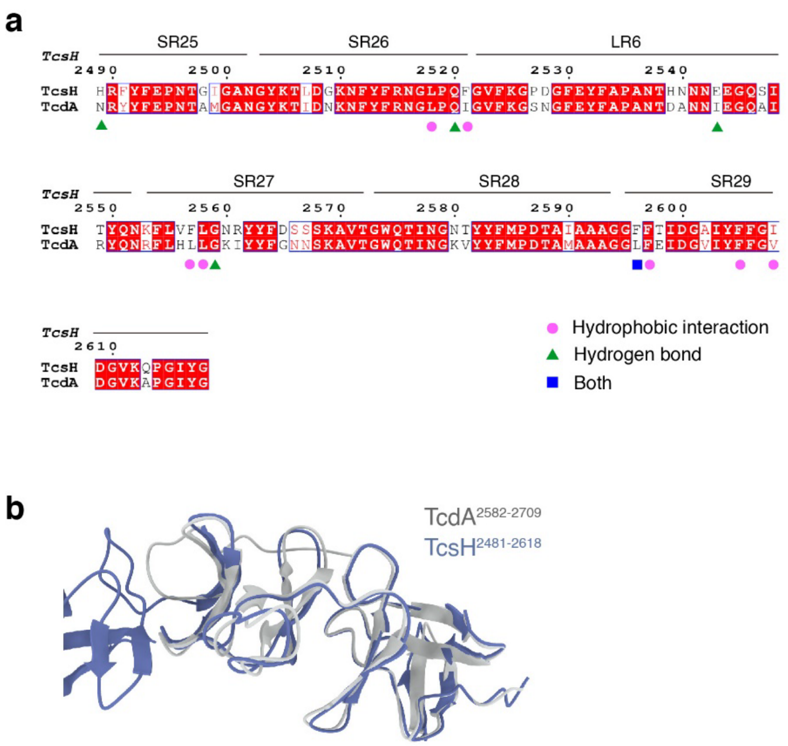

Supplementary Fig. 9 | Sequence and structural comparisons between TcsH CROP unit-VI and TcdA CROP unit-VII. a Amino acid sequence alignment between TcsH CROP unit-VI and TcdA CROP unit-VII. The LRs and SRs of TcsH are labeled on the top. Invariable residues are colored red. Key residues contribute to the hydrophobic interaction, hydrogen bonds or both are labeled as pink circles, green triangles, and blue squares, respectively. **b** Superimposed structure of TcsH$^{2236\text{-}2618}$ (dark blue) and TcdA$^{2582\text{-}2709}$ (grey; PDB: 2F6E).

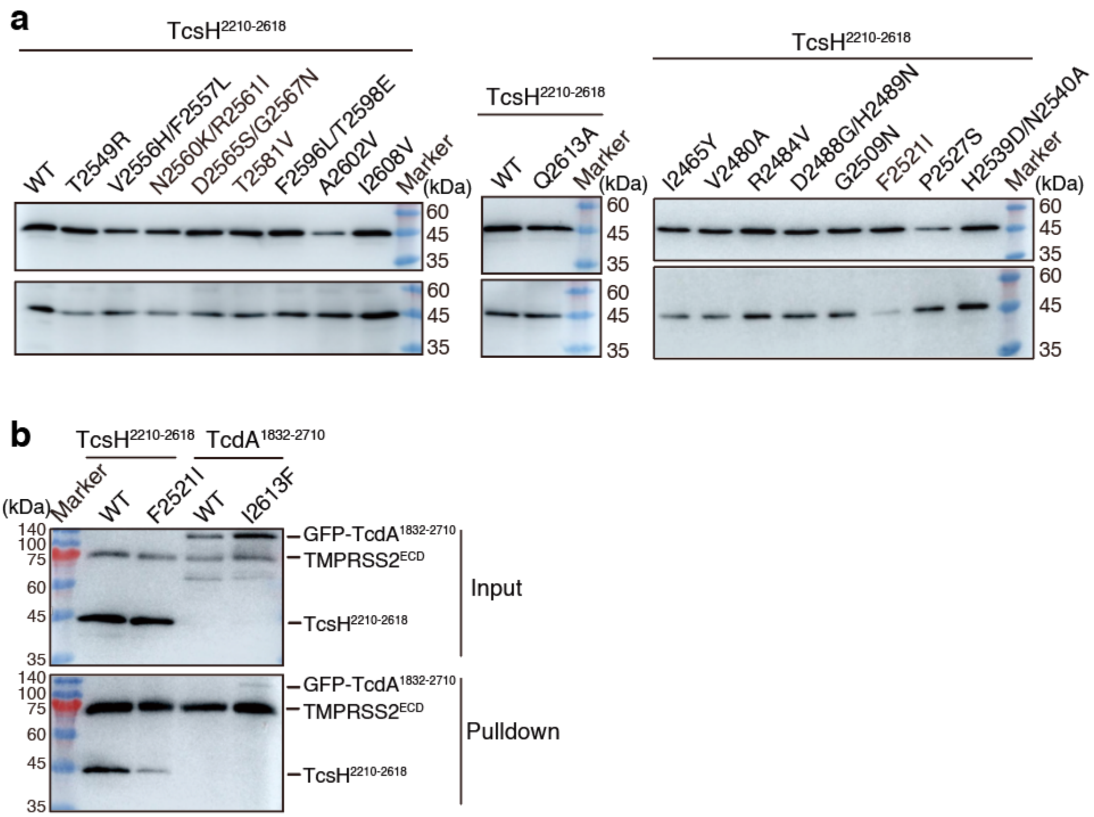

Supplementary Fig. 10│Characterization of binding of TcsH$^{2210\text{-}2618}$ variants to TMPRSS2^{ECD}. a The binding of TcsH$^{2210\text{-}2618}$ mutants to the Protein A resin immobilized Fc-tagged TMPRSS2^{ECD} was examined using pull-down assays. Samples were analyzed by immunoblot. Site-direct point mutations were designed based on varied residues between TcsH CROP unit-VI and homolog fragments in TcdA. **b** The binding of a TcdA$^{1832\text{-}2710}$ mutant (I2613F) to the Protein A resin immobilized Fc-tagged TMPRSS2^{ECD} was examined using pull-down assays. Samples were analyzed by immunoblot. Source data are provided as a Source Data file.

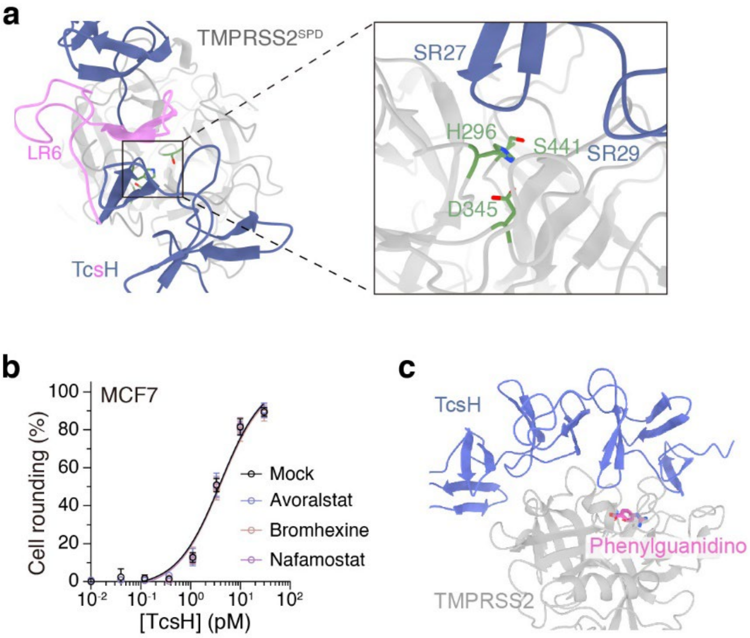

Supplementary Fig. 11 | TcsH covers the catalytic pocket of TMPRSS2. a In the top view of the TcsH-TMPRSS2 interface, loops of TcsH SR27 and SR29 stretch into the catalytic pocket of TMPRSS2. **b** TMPRSS2 inhibitors Avoralstat, Bromhexine, and Nafamostat fail to protect the MCF7 cells from TcsH. The MCF7 cells were pretreated with or without 10 μM Avoralstat, Bromhexine, or Nafamostat for 4 hours and then exposed to different concentrations of TcsH for 3 hours. Percentages of rounded cells were quantified and plotted on the chart. data are shown as mean ± SD. **c** The phenylguanidino moiety (magenta) covalently bound to the catalytic serine residue of TMPRSS2 (grey) and buried deep in the catalytic pocket of TMPRSS2, do not interfere the binding of TcsH (dark blue) with TMPRSS2. Source data are provided as a Source Data file.

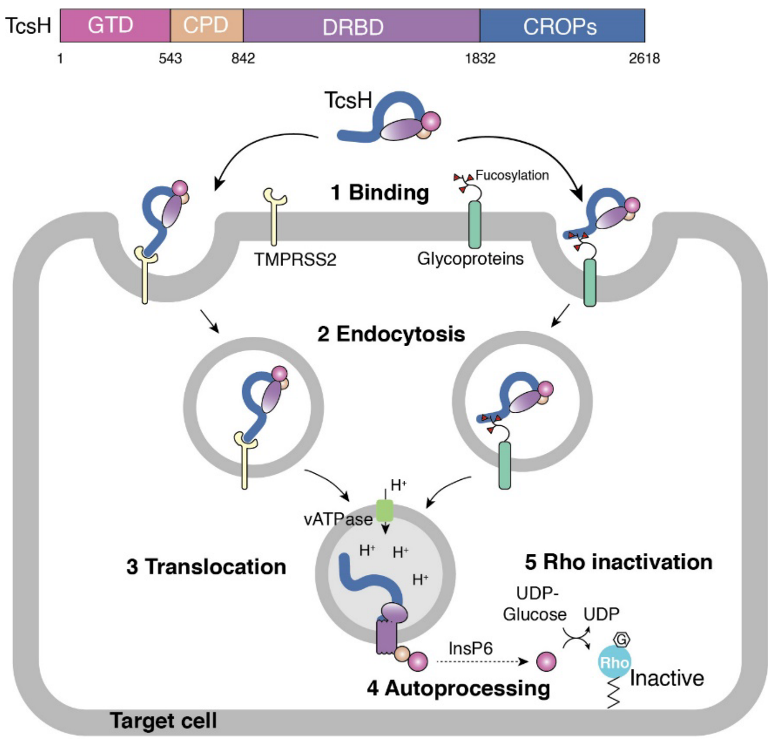

Supplementary Fig. 12 | Scheme of toxin action of TcsH. TcsH binds to the extracellular domain of TMPRSS2 or fucosylated glycans on glycoproteins via its C-terminal half CROPs. Then the toxin-receptor complex is endocytosed. In the acidified endosomes, TcsH inserts into the endosomal membrane and translocates the GTD and CPD into the cytosol. In the cytosol, CPD is activated by inositol hexakisphosphate (InsP6) and releases GTD. GTD glucosylates and inactivates small GTPase proteins like Rac1 and Rho to cause cytopathic effects and cell death.

Supplementary Table

Supplementary Table 1. Statistics of 3D reconstructions and model refinement.

	TcsH-TMPRSS2	TcsH$^{2236-2618}$-TMPRSS2
**Data collection**		
EM equipment	FEI Titan Krios	
Voltage (kV)	300	
Detector	K3	
Magnification	81,000	
Pixel size (Å)	1.087	
Electron dose (e-/Å^2)	50	
Defocus range (μm)	1.5~2.0	
**Reconstruction**		
Software	CryoSPARC	
Symmetry	C1	
Number of particles	87,378	760,140
Final masked resolution (Å)	3.2	3.0
Map sharpening B-factor (Å^2)	-103.7	-153.3
EMDB code	EMD-36301	EMD-36303
**Model building**		
Software	Coot-0.8.9/Chimera	
Refinement	Phenix	
Protein residues	2,955	498
B factors (Å^2)	73.9	45.3
PDB code	8JHZ	8JI0
**Validation**		
R.m.s deviations		
Bonds length (Å)	0.007	0.003
Bonds Angle (°)	0.639	0.594
Ramachandran plot statistics (%)		
Favored	94.91	90.45
Allowed	4.85	8.94
Outlier	0.24	0.61
Clashscore	9.04	10.06
CaBLAM outliers (%)	2.2	4.9
MolProbity score	2.26	2.65
